# Circ_0084582 Facilitates Cell Growth, Migration, Invasion, and Angiopoiesis in Osteosarcoma *via* Mediating the miR-485-3p/JAG1 Axis

**DOI:** 10.3389/fgene.2021.690956

**Published:** 2021-08-05

**Authors:** Peng Gao, Xincheng Zhao, Keying Yu, Ziqiang Zhu

**Affiliations:** ^1^Department of Orthopedics Surgery, The Second Hospital of Xuzhou Coal Mining Group, Xuzhou, China; ^2^Department of Orthopedics Surgery, The General Hospital of Xuzhou Coal Mining Group, Xuzhou, China

**Keywords:** circ_0084582, osteosarcoma, miR-485-3p, JAG1, progression

## Abstract

Osteosarcoma (OS) is the most representative bone cancer, and circular RNAs serve as pivotal regulators in the progression of OS. This research was designed to explore the role and functional mechanism of circ_0084582 in OS. Circ_0084582, microRNA-485-3p (miR-485-3p), and Jagged1 (JAG1) levels were measured by quantitative real-time polymerase chain reaction. Cell proliferation was examined via 3-(4, 5-dimethylthiazol-2-y1)-2, 5-diphenyl tetrazolium bromide (MTT) assay. Cell cycle progression was analyzed by flow cytometry. Wound healing and transwell assays were performed for evaluating cell migration and invasion. Angiopoiesis was assessed using the tube formation assay. Protein detection was conducted using Western blot. The target relation was identified by the dual-luciferase reporter assay, RNA immunoprecipitation (RIP) assay, and RNA pull-down assay. A xenograft experiment was applied for analyzing the effect of circ_0084582 on OS *in vivo*. Circ_0084582 was highly expressed in OS tissues and cells. Circ_0084582 knockdown reduced cell proliferation, cell cycle progression, migration, invasion, and angiopoiesis of OS cells. JAG1 was upregulated in OS, and its overexpression reversed the effects of circ_0084582 knockdown on OS cells. Circ_0084582 targeted miR-485-3p, and miR-485-3p targeted JAG1, and circ_0084582 could affect the JAG1 level by sponging miR-485-3p. The function of circ_0084582 in OS progression was also achieved by sponging miR-485-3p. Circ_0084582 knockdown decreased OS growth *in vivo* partly by the miR-485-3p–mediated JAG1 downregulation. These results indicate that circ_0084582 functions as a tumorigenic factor in OS via the regulation of miR-485-3p/JAG1 axis.

## Highlights

- Knockdown of circ_0084582 represses cell growth, migration, invasion, and angiopoiesis of osteosarcoma cells.
- JAG1 overexpression or miR-485-3p inhibition reverses the function of circ_0084582 downregulation in the progression of osteosarcoma.
- Circ_0084582 sponges miR-485-3p to regulate the expression of JAG1.

## Introduction

Osteosarcoma (OS) has become the most common primary bone carcinoma around the world (Lilienthal and Herold, 2020). Radiation exposure and alkylating agents are the major risk factors for OS, and rapid bone proliferation is also associated with OS (Ritter and Bielack, 2010). The therapeutic management of OS has been greatly improved in recent years, such as surgery, chemotherapy, stem cell therapy, and pharmacogenomics (Yan et al., 2016; Gianferante et al., 2017). However, tumor metastasis remains a huge challenge in the treatment and prognosis of OS (Yang et al., 2020). Therefore, it is essential to enhance the understanding of the molecular pathogenetic mechanism of OS and discover novel molecular targets.

Circular RNAs (circRNAs) are special single-stranded RNA molecules with covalently closed loops, and the dysregulation of circRNA is vital for the initiation and progression of OS (Li et al., 2021). CircRNAs act as the regulatory elements of genes through the “sponge-like” function of microRNAs (miRNAs) (Panda, 2018). For example, circ_0002137 promotes cell invasion and cell cycle progression in OS by sponging miR-433-5p to modulate the IGF1R level (Zhang et al., 2021). Circ_0056285 regulates OS cell apoptosis and proliferation by targeting the miR-1244/TRIM44 axis (Huo and Dou, 2021). Li et al. (2020) prove that circ_0000073 contributes to the development of OS by upregulating NRAS via the sponge effects on miR-145-5pa and miR-151-3p. Circ_0084582 is also shown to be upregulated in OS samples (Li et al., 2020). However, no research focuses on the function and mechanism of circ_0084582 in OS.

MicroRNAs can degrade mRNAs and inhibit the translation by targeting the 3′-untranslated regions (3′-UTRs) (Matoulkova et al., 2012). The differential expression of miRNAs is also correlated with the malignant progression of OS, and miRNAs have improved the diagnosis and treatment of OS (Wang et al., 2019; Zhang et al., 2019). The MiR-485-3p level was decreased in OS tissues, and the oncogenic process of OS was controlled by miR-485-3p via targeting CTBP1 (Du et al., 2018). It is not clear whether circ_0084582 has the sponge effect on miR-485-3p in OS.

The Notch signal pathway is involved in tumor biology and development (Xiao et al., 2016). Jagged1 (JAG1) is one of the representative ligands for Notch receptors to activate the Notch pathway. The previous study also affirms that JAG1 is a pro-oncogene in OS (Hu et al., 2017), and miR-26a repressed tumor growth by targeting JAG1 in OS (Lu et al., 2017). Herein, we investigate the target relation between miR-485-3p and JAG1 in OS.

This research explores the role of circ_0084582 in OS progression and its regulatory effect on JAG1 by targeting miR-485-3p. The presumptive signal network circ_0084582/miR-485-3p/JAG1 was ascertained after a series of experiments.

## Materials and Methods

### Tissue Collection

Thirty patients with OS were recruited into The Second Hospital of Xuzhou Coal Mining Group and received the surgical resection. Thirty paired OS tissues and the normal adjacent tissues were acquired after the surgery and preserved at −80°C. This study was carried out after obtaining written informed consent from patients and ratification from the Ethics Committee of The Second Hospital of Xuzhou Coal Mining Group.

### Cell Culture and Transfection

In this study, cell lines (COBIOER, Nanjing, China) were all cultivated in a 37°C, 5% CO_2_ humidified incubator. Normal osteoblast cell line hFOB 1.19 was maintained in F12/Dulbecco’s modified Eagle’s medium (DMEM) (1:1) containing 0.3 mg/mL G418 and 2.5 mM L-glutamine. OS cell lines MG63 and U2OS were, respectively, sustained in DMEM and McCoy’s 5A medium. Ten percent fetal bovine serum (FBS) and 1% penicillin/streptomycin were supplemented into the media. The used reagents were bought from Gibco (Carlsbad, CA, United States).

Lentiviral vectors (GenePharma, Shanghai, China) containing short hairpin RNA (shRNA) of circ_0084582 (sh-circ_0084582-1, sh-circ_0084582-2, sh-circ_0084582-3) were applied for the stable transfection, and sh-NC was used as the negative control (NC). The pcDNA expression vector and the pcDNA-JAG1 vector (JAG1) were obtained from Genewiz (Suzhou, China). The mimics and inhibitors of miRNA NC (miR-NC, anti-NC) and miR-485-3p (miR-485-3p, anti-miR-485-3p) were synthesized by RIBOBIO (Guangzhou, China). Lipofectamine3000 reagent (Invitrogen, Carlsbad, CA, United States) was applied for cell transfection following the detailed procedures provided by the producer.

### The Quantitative Real-Time Polymerase Chain Reaction

As previously described, the SYBR Green PCR Kit and ABI Prism 7500 sequence detection system (Applied Biosystems, Foster City, CA, United States) were exploited for quantitative real-time polymerase chain reaction (qRT-PCR) analysis (Jin et al., 2019). The relative expression analysis was performed using the 2^–ΔΔ*Ct*^ method. Glyceraldehyde-3-phosphate dehydrogenase (GAPDH) and U6 were selected as the reference genes for circ_0084582/JAG1 and miR-485-3p (Livak and Schmittgen, 2001). The primer (forward, F; reverse, R) sequences of each molecule are listed as follows: circ_0084582 (F: 5′-ACCACCAGTCTTCACCTCCA-3′, R: 5′-CTGCAGTGTCAGGCAAAGTC-3′); miR-485-3p (F: 5′-GCCG AGGUCAUACACGGCUCU-3′, R: 5′-CTCAACTGGTGTCGTG GA-3′); JAG1 (F: 5′-CTCATCAGCGGTGTCTCAAC-3′, R: 5′-GGCACACACACTTAAATCCG-3′); GAPDH (F: 5′-GGGAG CCAAAAGGGTCATCA-3′, R: 5′-TGATGGCATGGACTGT GGTC-3′); U6 (F: 5′-ATTGGAACGATACAGAGAAGATT-3′, R: 5′-GGAACGCTTCACGAATTTG-3′). In addition, the stability of circ_0084582 was detected using qRT-PCR after the incubation of RNase R (GENESEED, Guangzhou, China) in total RNA, and the localization of circ_0084582 was analyzed via qRT-PCR after the nuclear or cytoplasmic RNA isolation using PARIS^TM^ Kit (Invitrogen).

### 3-(4, 5-Dimethylthiazol-2-y1)-2, 5-Diphenyl Tetrazolium Bromide Assay

MG63 and U2OS cells were inoculated into 96-well plates overnight with 2 × 10^3^ cells per well. Every day post-transfection, 20 μL/well 3-(4, 5-dimethylthiazol-2-y1)-2, 5-diphenyl tetrazolium bromide (MTT) (Invitrogen) was instilled into the well-plates to incubate with cells for 4 h. Then, the supernatant of each well was discarded, and formazan was dissolved with dimethyl sulfoxide (DMSO; Invitrogen) for 10 min. Whereafter, the optical density value was analyzed at 490 nm under the microplate reader.

### Cell Cycle Assay

The progression of the cell cycle was assessed *via* a Cell Cycle Analysis kit (Beyotime, Shanghai, China) in compliance with the manufacturer’s instruction book. Then, cell proportion was measured at each phase through a flow cytometer (BD Biosciences, San Diego, CA, United States).

### Wound Healing Assay

The six-well plates were planted with 2 × 10^5^ cells overnight, and we created two straight scratches in each well using a sterile pipette tip. Phosphate buffer solution (PBS; Gibco) was added to wash the scraped cells, and the remaining cells in the wells were incubated with normal cell medium for 24 h. Cells were photographed and the wound width was measured at 0 h (a) and 24 h (b). Wound healing ratio [(a − b)/a × 100%] was used to evaluate the migratory capacity.

### Transwell Assay

The transwell 24-well chamber (Corning Life Sciences, Corning, NY, United States) was employed for the measurement of migration and invasion in MG63 and U2OS cells. Before the invasion assay, the upper chamber must be covered with matrigel (Corning Life Sciences). The upper and lower chambers were, respectively, added with cell suspension in serum-free medium and medium with 10% FBS; 48 h later, cells in the lower chamber were fixed and dyed with methanol and crystal violet (Sangon Biotech, Shanghai, China). Instantly, the stained cells were counted via a microscope.

### Tube Formation Assay

Angiopoiesis was examined using tube formation assay. The supernatants of MG63 and U2OS cells were collected and co-incubated with human umbilical vein endothelial cells (HUVECs; COBIOER) in 96-well plates coated with matrigel (BD Bioscience). After cell culture for 48 h, the capillary-like branches were counted in three random fields of view under the computer-assisted microscope.

### Western Blot

First, total proteins were isolated using radioimmunoprecipitation assay (RIPA) buffer (Abcam, Cambridge, MA, United States), and 40 μg proteins were loaded onto 10% sodium dodecyl sulfate-polyacrylamide gel for 90 min. Then, protein transferring to polyvinylidene fluoride membranes (Millipore, Bedford, MA, United States) was carried out for 1 h and protein blocking with non-fat dry milk (Beyotime) was conducted for 3 h. Subsequently, PVDF membranes were incubated with the primary antibodies and secondary antibody (Abcam, ab205718, 1:5000). The primary antibodies included JAG1 (Abcam, ab7771, 1:1000), vascular endothelial growth factor (VEGFA; Abcam, ab46154, 1:1000), c-myc (Abcam, ab32072, 1:1000), and beta-actin (β-actin; Abcam, ab8227, 1:3000). Then, the combined signals were detected by the enhanced chemiluminescence kit (Millipore), followed by data analysis through the Image J software (NIH, Bethesda, MD, United States).

### Dual-Luciferase Reporter Assay

Bioinformatics analysis between miR-485-3p and circ_0084582 or JAG1 was performed using circinteractome^1^ and miRDB.^2^ Then wild-type (WT) luciferase reporters were constructed through cloning the sequences of WT circ_0084582 and JAG1 3′UTR into the pmirGLO luciferase vector (Promega, Madison, WI, United States). These positive vectors were named as WT-circ_0084582 and WT-JAG1 3′UTR. Similarly, the mutant (MUT) reporters MUT-circ_0084582 and MUT-JAG1 3′UTR- were obtained after the binding sites of miR-485-3p in the sequence of circ_0084582 and JAG1 3′UTR were mutated. Whereafter, the above reporters were respectively co-transfected with miR-485-3p or miR-NC into MG63 and U2OS cells. The dual-luciferase reporter assay system (Promega) was applied to determine the relative luciferase activities of harvested cells.

### RNA Immunoprecipitation Assay

The potential target binding was analyzed using Magna RIP RNA-Binding Protein Immunoprecipitation Kit (Millipore). In brief, the lysates of MG63 and U2OS cells were incubated with the antibody-conjugated magnetic beads of the Argonaute 2 (Ago2) or Immunoglobulin G (IgG) group at 4°C overnight. The immunoprecipitated RNA was extracted, and the expression analysis (circ_0084582, miR-485-3p and JAG1) was performed by qRT-PCR.

### RNA Pull-Down Assay

MG63 and U2OS cells were, respectively, transfected with biotinylated miRNA mimics (RIBOBIO). MUT-bio-miR-485-3p and bio-NC were used as the control groups for WT-bio-miR-155 group. Cells were harvested after 48 h and detected via Pierce^TM^ Magnetic RNA-Protein Pull-Down Kit (Thermo Fisher Scientific, Waltham, MA, United States). Finally, the total RNA was purified for the qRT-PCR detection of circ_0084582 and JAG1.

### Xenograft Tumor Assay

Twelve BALB/c male nude mice were obtained from Vital River Laboratory Animal Technology (Beijing, China). These mice were arbitrarily divided into two groups with six mice per group. Then sh-circ_0084582-1 or sh-NC transfected MG63 cells (2 × 10^6^ cells/mice) were subcutaneously injected into mice dorsum. The tumor volume (length × width^2^ × 0.5) was measured every 4 days. After cell injection for 28 days, all mice were sacrificed by the CO_2_ asphyxia method. Mice were dissected and tumors were weighed on an electronic scale, followed by the detection of circ_0084582 via qRT-PCR and protein analysis of JAG1 or Ki67 using an immunohistochemistry (IHC) assay (Chen et al., 2020). This animal experiment has obtained permission from the Animal Ethics Committee of The Second Hospital of Xuzhou Coal Mining Group. All protocols were performed according to the ARRIVE guidelines and the Basel Declaration.

### Statistical Analysis

The data were exhibited as mean ± standard deviation (SD), then SPSS 19.0 (SPSS Inc., Chicago, IL, United States) and GraphPad Prism 7 (GraphPad Inc., La Jolla, CA, United States) were exploited for data analysis. The linear relation was analyzed in OS tissues using Pearson’s correlation coefficient. The analysis of difference was administrated through Student’s *t*-test one-way analysis of variance (ANOVA) followed by Tukey’s test. *P* < 0.05 represented a significant difference statistically.

## Results

### High Expression of circ_0084582 Was Found in OS Tissues and Cells

**FIGURE 1:**
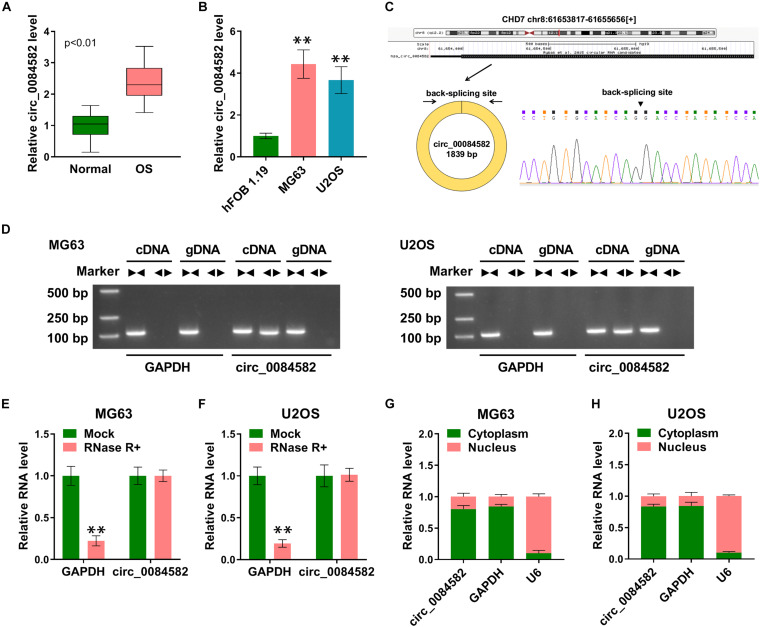
High expression of circ_0084582 is found in OS tissues and cells. **(A,B)** The expression of circ_0084582 is examined by qRT-PCR in OS tissues **(A)** and cells **(B)**. **(C)** The back-splicing bites of circ_0084582. **(D)** Circ_0084582 and linear GAPDH are distinguished by gel electrophoresis. **(E,F)** GAPDH and circ_0084582 levels are examined by qRT-PCR in RNase R and Mock groups. **(G,H)** The qRT-PCR is used for the detection of circ_0084582, GAPDH, and U6 in cytoplasm and nucleus. All experiments were performed with three biological replications. ^∗∗^*P* < 0.01.

The expression of circ_0084582 in OS was first detected by qRT-PCR. The results revealed that the circ_0084582 level was higher in OS tissues than that in normal tissues (Figure 1A). By comparison with normal hFOB 1.19 cells, circ_0084582 was also displayed to be upregulated in MG63 and U2OS cells (Figure 1B). The back-splicing sites of circ_0084582 are shown in Figure 1C, and its sequence length was 1839 bp. Gel electrophoresis was performed to identify the presence of circRNA using convergent and divergent primers. The images indicate that linear GAPDH was not detected by divergent primers with cDNA and gDNA as the templates, and circ_0084582 was detected by divergent primers in cDNA (Figure 1D). Circ_0084582 was more resistant to RNase R relative to the linear GAPDH, suggesting that circ_0084582 was highly stable (Figures 1E,F). Additionally, the expression distribution in the nucleus and cytoplasm demonstrate that circ_0084582 was localized in the cytoplasm of OS cells (Figure 1G,H). Circ_0084582 was identified as an upregulated circRNA in OS.

### Knockdown of circ_0084582 Impeded Cell Proliferation and Cell Cycle Progression, Migration, Invasion, and Angiopoiesis in OS Cells

**FIGURE 2:**
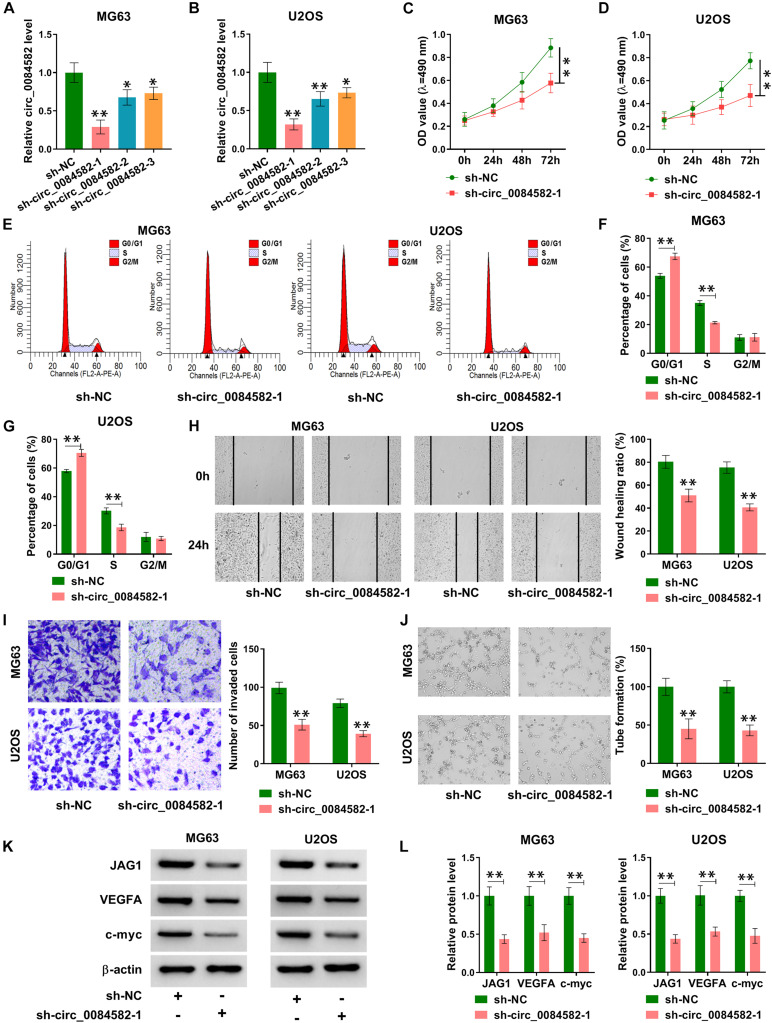
Knockdown of circ_0084582 impedes cell proliferation, cell cycle progression, migration, invasion, and angiopoiesis in OS cells. **(A,B)** The knockdown effects of sh-circ_0084582-1, sh-circ_0084582-2, and sh-circ_0084582-3 on the circ_0084582 expression are determined using qRT-PCR in OS cells. **(C,D)** MTT was used for detecting cell proliferation after MG63 and U2OS cells were transfected with sh-circ_0084582-1 or sh-NC. **(E–G)** Flow cytometry was used for analyzing the progression of cell cycle. **(H,I)** Transwell assay was used for evaluating cell migration **(H)** and invasion **(I)**. **(J)** Tube formation assay was used for assessing angiopoiesis. **(K,L)** Western blot was used for assaying the protein levels of JAG1, VEGFA, and c-myc. All experiments were performed with three biological replications. ^∗^*P* < 0.05, ^∗∗^*P* < 0.01.

The role of circ_0084582 in OS was investigated through cellular experiments. The shRNA transfection was used to knock down the expression of circ_0084582, and the interference efficiencies were great (Figures 2A,B). The following assays were performed using sh-circ_0084582-1 with the most significant knockdown for circ_0084582. MTT assay and flow cytometry showed that the downregulation of circ_0084582 reduced cell proliferation (Figures 2C,D) and cell cycle progression (Figures 2E–G) in MG63 and U2OS cells. By performing wound-healing and transwell assays, we found that cell migration (Figure 2H) and invasion (Figure 2I) were suppressed by the silence of circ_0084582. A tube formation assay revealed that the angiopoietic ability was decreased in sh-circ_0084582-1 group in contrast with the sh-NC group (Figure 2J). Western blot analysis manifested that transfection of sh-circ_0084582-1 evoked the protein expression downregulation of JAG1, VEGFA (an angiopoietic marker), and c-myc (cell proliferation) compared with transfection of sh-NC (Figures 2K,L). Collectively, circ_0084582 knockdown inhibited the progression of OS, and its function might be related to the regulation of JAG1.

### JAG1 Was Overexpressed in OS Samples and Cells

**FIGURE 3:**
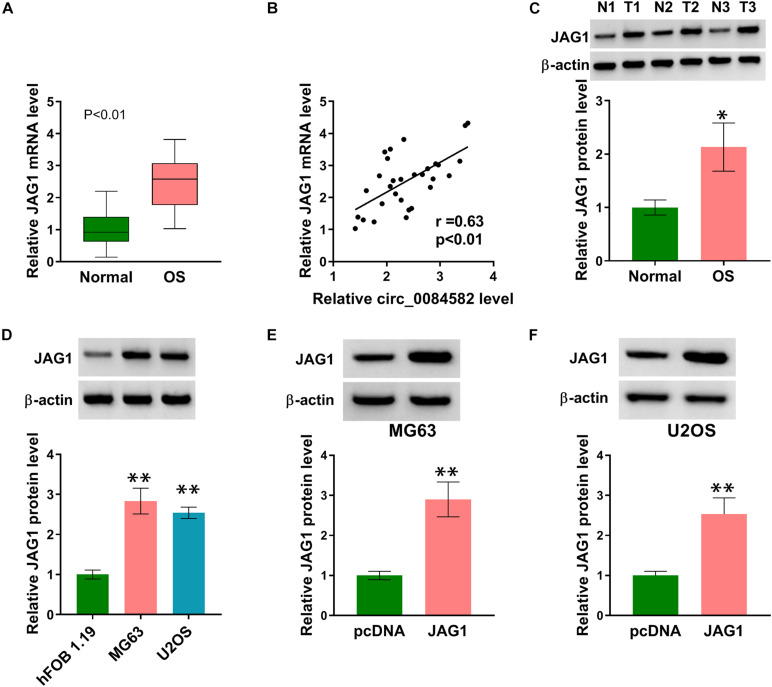
JAG1 was overexpressed in OS samples and cells. **(A)** The mRNA level of JAG1 was quantified via qRT-PCR in OS samples. **(B)** The linear relationship between circ_0084582 and JAG1 was analyzed via Pearson’s correlation coefficient. **(C,D)** JAG1 protein expression was measured via Western blot in OS samples **(C)** and cells **(D)**. **(E,F)** The transfection efficiency of JAG1 was assessed via Western blot in MG63 and U2OS cells. All experiments were performed with three biological replications. ^∗^*P* < 0.05, ^∗∗^*P* < 0.01.

Subsequently, we performed expression detection of JAG1 in OS tissues. As depicted in Figure 3A, the JAG1 mRNA level was much higher in OS samples than that in normal samples. Pearson’s correlation coefficient showed a positive relation (*r* = 0.63, *p* < 0.01) between the circ_0084582 level and JAG1 mRNA expression (Figure 3B). Consistently, the protein level of JAG1 was also increased in OS tissues by contrast with normal tissues (Figure 3C). In addition, Western blot also confirmed the upregulation of JAG1 in MG63 and U2OS cells relative to hFOB 1.19 cells (Figure 3D). JAG1 protein expression was enhanced after transfection of JAG1 in comparison with pcDNA group, indicating that JAG1 was successfully overexpressed by the JAG1 vector in MG63 and U2OS cells (Figures 3E,F). Thus, JAG1 was also validated to be upregulated in OS.

### JAG1 Overexpression Reversed the Effects of circ_0084582 Knockdown on OS Cells

**FIGURE 4:**
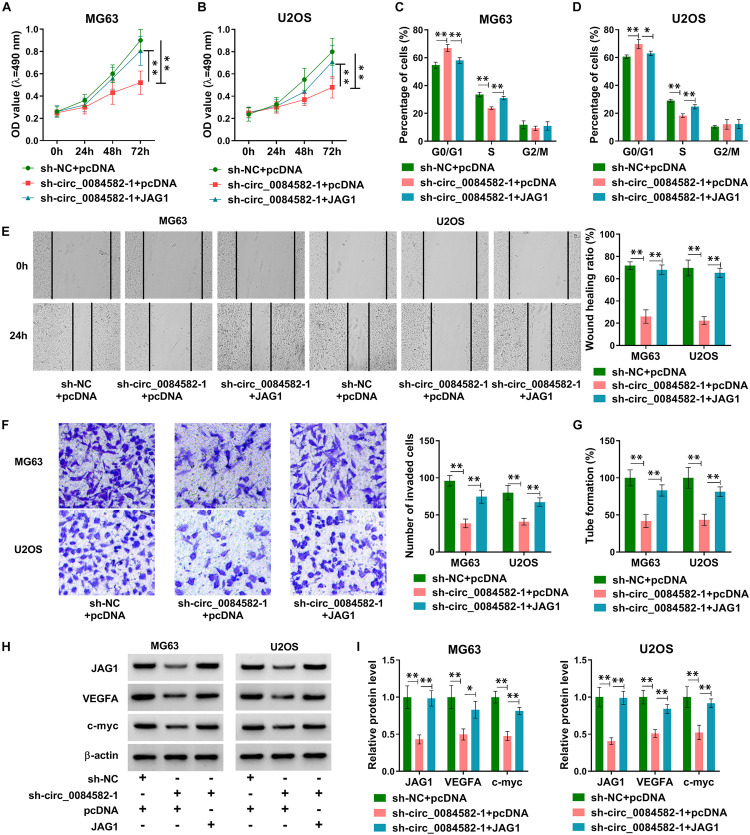
JAG1 overexpression reverses the effects of circ_0084582 knockdown on OS cells. **(A,B)** Cell proliferation was determined by MTT assay after transfection of sh-NC + pcDNA, sh-circ_0084582-1 + pcDNA, or sh-circ_0084582-1 + JAG1. **(C,D)** Cell cycle progression was analyzed by flow cytometry. **(E,F)** Cell migration and invasion were, respectively, measured using wound-healing and transwell assays. **(G)** Angiopoiesis was detected by a tube formation assay. **(H,I)** JAG1, VEGFA, and c-myc protein levels were examined by Western blot. All experiments were performed with three biological replications. ^∗^*P* < 0.05, ^∗∗^*P* < 0.01.

To explore whether JAG1 was related to the function of circ_0084582, MG63 and U2OS cells were transfected with sh-NC + pcDNA, sh-circ_0084582-1 + pcDNA, or sh-circ_0084582-1 + JAG1. The experimental results exhibit that sh-circ_0084582-1–induced cell proliferation inhibition (Figures 4A,B) and cell cycle arrest (Figures 4C,D) were abolished by transfection of JAG1. Also, the repressive influences of sh-circ_0084582-1 on cell migration (Figure 4E), invasion (Figure 4F), and angiopoiesis (Figure 4G) were all partly lightened following the overexpression of JAG1. The protein levels of JAG1, VEGFA, and c-myc were promoted in the sh-circ_0084582-1 + JAG1 group compared with sh-circ_0084582-1 + pcDNA group (Figures 4H,I). The above data demonstrate that JAG1 overexpression relieved the antitumor function of circ_0084582 downregulation in OS progression.

### Circ_0084582 Regulated the JAG1 Expression by Sponging miR-485-3p

**FIGURE 5:**
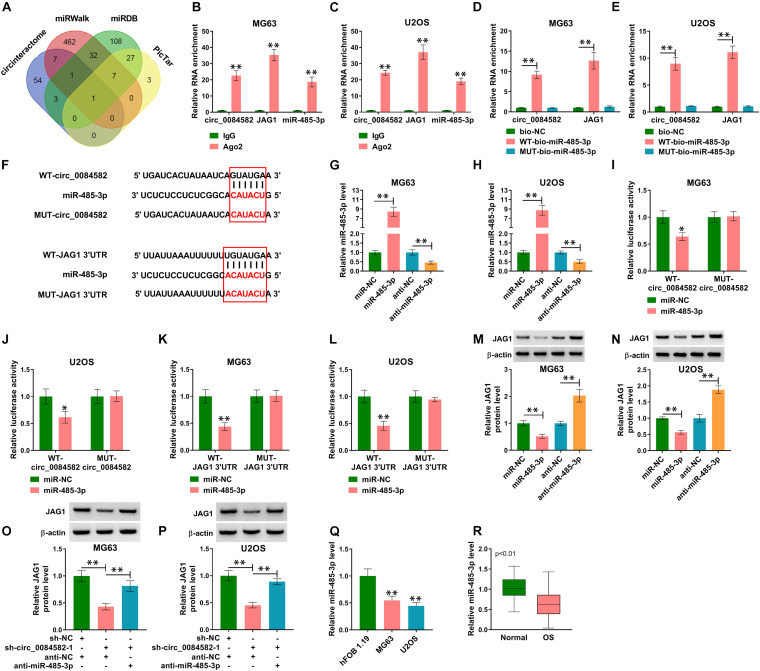
Circ_0084582 regulated the JAG1 expression by sponging miR-485-3p. **(A)** The mutual miRNA in target prediction for circ_0084582 and JAG1 was selected by Venn diagram analysis. **(B–E)** The potential binding between miR-485-3p and circ_0084582 or JAG1 was analyzed by RIP **(B,C)** and pull-down assays **(D,E)**. **(F)** Circinteractome and miRDB, respectively, show the binding sites between miR-485-3p and circ_0084582 or JAG1. **(G,H)** The efficiencies of miR-485-3p mimic and inhibitor were assessed by qRT-PCR in MG63 and U2OS cells. **(I–L)** The dual-luciferase reporter assay was used to affirm the interaction between miR-485-3p and circ_0084582 **(I,J)** or JAG1 **(K,L)**. **(M,N)** The effects of miR-485-3p overexpression or downregulation on JAG1 expression were analyzed via Western blot. **(O,P)** JAG1 protein level was assayed using Western blot after MG63 and U2OS cells were transfected with sh-NC, sh-circ_0084582-1, sh-circ_0084582-1 + anti-NC, or sh-circ_0084582-1 + anti-miR-485-3p. **(Q,R)** The miR-485-3p expression quantification was conducted using qRT-PCR in OS cells **(Q)** and tissues **(R)**. All experiments were performed with three biological replications. ^∗^*P* < 0.05, ^∗∗^*P* < 0.01.

Through the target prediction and Venn diagram analysis, only miR-485-3p was found to have binding sites with circ_0084582 and JAG1 concurrently (Figure 5A). The RIP assay demonstrated that circ_0084582, JAG1, and miR-485-3p were abundantly detected in the Ago2 group relative to the IgG group (Figures 5B,C). The pull-down assay also manifested that circ_0084582 and JAG1 could be captured by miR-485-3p in MG63 and U2OS cells (Figures 5D,E). The binding sites between miR-485-3p and circ_0084582 or JAG1 are shown in Figure 5F. The miR-485-3p overexpression and inhibition mediated by miR-485-3p and anti-miR-485-3p were excellent in MG63 and U2OS cells (Figures 5G,H). Furthermore, the dual-luciferase reporter assay suggests that miR-485-3p overexpression resulted in the inhibition of luciferase activity of the WT-circ_0084582 (Figures 5I,J) and WT-JAG1 3′UTR groups (Figures 5K,L), but it could not affect the luciferase signals of their MUT controls. Protein detection showed that JAG1 was downregulated after transfection of miR-485-3p, but it was upregulated by anti-miR-485-3p (Figures 5M,N). Moreover, knockdown of circ_0084582 inhibited the protein level of JAG1, and the miR-485-3p inhibitor attenuated this effect (Figures 5O,P). As for the expression of miR-485-3p in OS, our qRT-PCR data revealed that its expression was declined in OS cells (Figure 5Q) and tissues (Figure 5R) compared with the normal cell and tissues. Taken together, circ_0084582 could sponge miR-485-3p to regulate the JAG1 expression.

### Circ_0084582/miR-485-3p Axis Regulated the Malignant Progression of OS

**FIGURE 6:**
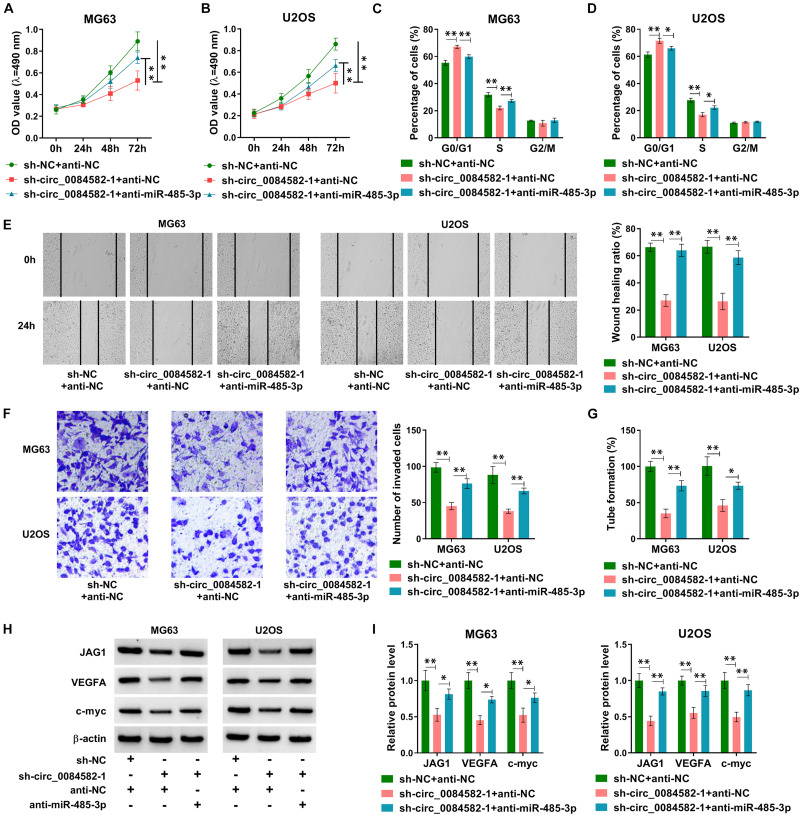
The Circ_0084582/miR-485-3p axis regulates the malignant progression of OS. **(A,B)** The detection of cell viability was carried out by MTT assay in sh-NC + anti-NC, sh-circ_0084582-1 + anti-NC, or sh-circ_0084582-1 + anti-miR-485-3p transfection groups. **(C,D)** The analysis of cell cycle progression was performed by flow cytometry. **(E,F)** The examination of cell migration **(E)** and invasion **(F)** was conducted by wound-healing and transwell assays. **(G)** The tube formation assay was applied for the measurement of angiopoiesis. **(H,I)** Western blot was used for the protein determination of JAG1, VEGFA, and c-myc. All experiments were performed with three biological replications. ^∗^*P* < 0.05, ^∗∗^*P* < 0.01.

The modulatory mechanism between circ_0084582 and miR-485-3p was investigated after transfection of sh-NC + anti-NC, sh-circ_0084582-1 + anti-NC, or sh-circ_0084582-1 + anti-miR-485-3p in MG63 and U2OS cells. Subsequent assays suggest that the introduction of anti-miR-485-3p mitigated those inhibitory effects on cell proliferation (Figures 6A,B), cell cycle progression (Figures 6C,D), migration/invasion (Figures 6E,F), and angiopoiesis (Figure 6G) caused by the knockdown of circ_0084582. Meanwhile, the sh-circ_0084582-1–induced downregulation of JAG1, VEGFA, and c-myc was reverted by miR-485-3p inhibition (Figures 6H,I). Overall, the involvement of circ_0084582 in OS progression was partly ascribed to the sponge effect on miR-485-3p.

### Circ_0084582 Regulated Tumor Growth of OS *in vivo* Partly by the miR-485-3p/JAG1 Axis

**FIGURE 7:**
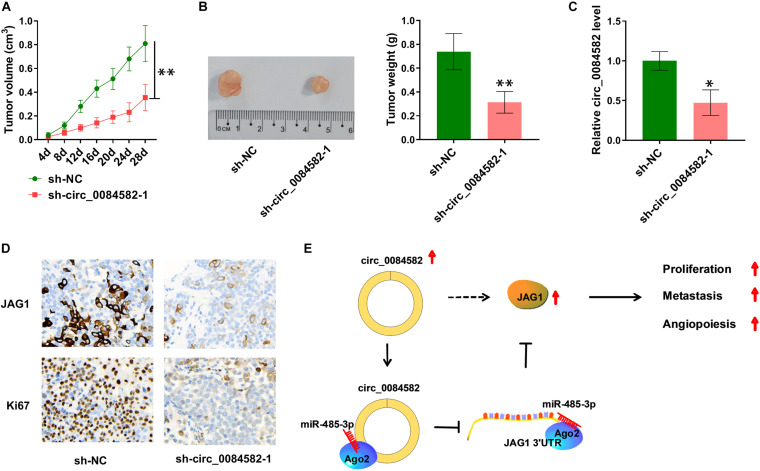
Circ_0084582 regulates tumor growth of OS *in vivo* partly by the miR-485-3p/JAG1 axis. **(A)** Tumor volume of the sh-circ_0084582-1 or sh-NC groups was measured per 4 days. **(B)** Tumor tissues were photographed and weighed. **(C)** The circ_0084582 expression was detected by qRT-PCR in tumor tissues. **(D)** JAG1 and Ki67 protein levels in excised tissues were assayed via IHC analysis. **(E)** The graphical summary of this study. All experiments were performed with three biological replications. ^∗^*P* < 0.05, ^∗∗^*P* < 0.01.

The influence of circ_0084582 on OS *in vivo* was analyzed via the xenograft model. Tumor volume (Figure 7A) and weight (Figure 7B) were observed to be decreased in the sh-circ_0084582-1 group compared with the sh-NC group. Circ_0084582 expression reduction was found in tumor tissues of the sh-circ_0084582-1 group relative to those of the sh-NC group (Figure 7C). In addition, IHC analysis manifested that JAG1 and Ki67 protein levels were suppressed after the downregulation of circ_0084582 (Figure 7D). Circ_0084582 could promote OS growth *in vivo* through affecting the miR-485-3p–mediated JAG1 level. All in all, circ_0084582 acted as a miR-485-3p sponge to induce the upregulation of JAG1 to facilitate the malignant behaviors of OS cells (Figure 7E).

## Discussion

It is crucial to research the molecular mechanism during the developing process of OS and seek therapeutic biomarkers for OS treatment. Herein, we found that circ_0084582 served as a tumorigenic molecule in the developing process of OS by depending on the miR-485-3p/JAG1 axis. The circ_0084582/miR-485-3p/JAG1 network might provide a novel direction for molecular targeted therapy of OS.

Our qRT-PCR indicated that circ_0084582 expression was enhanced in OS, and this result is consistent with the previous study (Li et al., 2020). Increasing evidence manifests the important involvement of circRNA dysregulation in different types of human tumors. He et al. (2021) find the downregulation of circRNA_100395 in prostate cancer, and they clarified that cell metastasis was inhibited by circRNA_100395. Chen et al. (2021) attest that the level of circ_0001360 in cutaneous squamous cell carcinoma was decreased, and the overexpression of circ_0001360 repressed the carcinogenesis. In addition, circ_0006948 was highly expressed in esophageal carcinoma, and it aggravated the tumor progression (Yue et al., 2021). The circ_0000372 overexpression facilitated cell growth and metastasis of colorectal cancer (Liu et al., 2021). Through further functional assays, we find that silencing the expression of circ_0084582 could suppress the malignant behaviors including proliferation, cell cycle progression, migration/invasion, and angiopoiesis. Thus, circ_0084582 was identified as an oncogenic circRNA in the OS progression.

Jagged1 was exhibited to be overexpressed in OS tissues and cells, implying that JAG1 might participate in the development of OS. JAG1 has been considered as a tumorigenic gene in ovarian carcinoma, breast carcinoma, and multiple myeloma (Jia et al., 2018; Liu et al., 2020; Zohny et al., 2020). Recent studies in OS prove that JAG1 enhanced tumor growth and metastasis (Lu et al., 2017; Wen et al., 2017). More interestingly, our data reveal that the antitumor effects of circ_0084582 knockdown in OS were all recovered by overexpression of JAG1. In other words, the regulation of circ_0084582 in the progression of OS was attributed to the positive effect on JAG1 expression.

Furthermore, miR-485-3p was predicted as a mutual miRNA among the targets for circ_0084582 and JAG1. Circ_0084582 and JAG1 could interact with miR-485-3p via the Ago2 protein. The subsequent target assays also verified the interaction of miR-485-3p with circ_0084582 or JAG1 in OS cells. The JAG1 level was negatively affected by miR-485-3p, and circ_0084582 triggered the upregulation of JAG1 via targeting miR-485-3p. A variety of circRNA/miRNA/mRNA regulatory networks have emerged in tumor research. For instance, circ_0000527/miR-98-5p/XIAP facilitates the malignant progression of retinoblastoma cells (Yu et al., 2021), and circ_0018414/miR-6807-3p/DKK1 impedes the tumor development in lung adenocarcinoma (Yao et al., 2021). In this study, the circ_0084582/miR-485-3p axis is validated to regulate JAG1 expression and cellular processes in OS. Circ_0084582 is considered to function as a tumor promoter in OS through modulating the JAG1 level via sponging miR-485-3p. Additionally, *in vivo* experiments also manifest that circ_0084582 aggravates tumor growth by upregulating JAG1.

In conclusion, our results unraveled that circ_0084582 aggravates the tumorigenic phenotypes in OS cells via the regulation of the miR-485-3p/JAG1 pathway. The specific circ_0084582/miR-485-3p/JAG1 signaling network in OS progression was first unveiled, and it might contribute to the further exploration of the molecular pathogenesis in OS. In addition, circ_0084582 is likely to be used as a favorable diagnostic and therapeutic indicator in OS.

## Data Availability Statement

The raw data supporting the conclusions of this article will be made available by the authors, without undue reservation.

## Ethics Statement

The studies involving human participants were reviewed and approved by The Second Hospital of Xuzhou Coal Mining Group. The patients/participants provided their written informed consent to participate in this study. The animal study was reviewed and approved by The Second Hospital of Xuzhou Coal Mining Group.

## Author Contributions

PG designed the study. PG and XZ analyzed the data. KY performed the experiments. XZ and ZZ summarized the data and wrote the manuscript. All authors contributed to this study and read and approved the manuscript.

## Conflict of Interest

The authors declare that the research was conducted in the absence of any commercial or financial relationships that could be construed as a potential conflict of interest.

## Publisher’s Note

All claims expressed in this article are solely those of the authors and do not necessarily represent those of their affiliated organizations, or those of the publisher, the editors and the reviewers. Any product that may be evaluated in this article, or claim that may be made by its manufacturer, is not guaranteed or endorsed by the publisher.

## Footnotes

1
https://circinteractome.nia.nih.gov/

2
http://www.mirdb.org/
